# Supplementary material for: Identification and validation of a prognostic model based on immune-related genes in ovarian carcinoma
Source: PeerJ. 2024 Oct 31;12:e18235. doi: 10.7717/peerj.18235 (PMC11531744; doi:10.7717/peerj.18235)
Supplement: Supplemental Information 1 [file peerj-12-18235-s001.docx]

**The Minimum Information for Publication of Quantitative Real-Time PCR Experiments (MIQE) checklist for authors, reviewers, and editors (Bustin et al. 2009).^a^**

| Item to check | Importance | Checklist |
| --- | --- | --- |
| Experimental design |  |  |
| Definition of experimental and control groups | E | SM |
| Number within each group | E | SM |
| Assay carried out by core lab or investigator's lab? | D | ∅ |
| Acknowledgement of authors' contributions | D | ∅ |
| Sample |  |  |
| Description | E | SM |
| Volume/mass of sample processed | D | ∅ |
| Microdissection or macrodissection | E | SM |
| Processing procedure | E | SM |
| If frozen, how and how quickly? | E | SM |
| If fixed, with what and how quickly? | E | SM |
| Sample storage conditions and duration (especially for FFPE^b^ samples) | E | SM |
| Nucleic acid extraction |  |  |
| Procedure and/or instrumentation | E | SM |
| Name of kit and details of any modifications | E | SM |
| Source of additional reagents used | D | SM |
| Details of DNase or RNase treatment | E | SM |
| Contamination assessment (DNA or RNA) | E | SM |
| Nucleic acid quantification | E | SM |
| Instrument and method | E | SM |
| Purity (A260/A280) | D | ∅ |
| Yield | D | ∅ |
| RNA integrity: method/instrument | E | SM |
| RIN/RQI or Cq of 3’ and 5’ transcripts | E | ∅ |
| Electrophoresis traces | D | ∅ |
| Inhibition testing (Cq dilutions, spike, or other) | E | SM |
| Reverse transcription |  |  |
| Complete reaction conditions | E | SM |
| Amount of RNA and reaction volume | E | SM |
| Priming oligonucleotide (if using GSP) and concentration | E | SM |
| Reverse transcriptase and concentration | E | SM |
| Temperature and time | E | SM |
| Manufacturer of reagents and catalogue numbers | D | SM |
| Storage conditions of cDNA | D | ∅ |
| qPCR target information |  |  |
| Gene symbol | E | SM |
| Sequence accession number | E | SM |
| Amplicon length | E | SM |
| In silico specificity screen (BLAST, and so on) | E | ∅ |
| Location of each primer by exon or intron (if applicable) | E | ∅ |
| What splice variants are targeted? | E | ∅ |
| qPCR oligonucleotides |  |  |
| Primer sequences | E | SM |
| Location and identity of any modifications | E | ∅ |
| qPCR protocol |  |  |
| Complete reaction conditions | E | SM |
| Reaction volume and amount of cDNA/DNA | E | SM |
| Primer, (probe), Mg^2+^, and dNTP concentrations | E | SM |
| Polymerase identity and concentration | E | SM |
| Buffer/kit identity and manufacturer | E | SM |
| Additives (SYBR Green I, DMSO, and so forth) | E | SM |
| Complete thermocycling parameters | E | SM |
| Manufacturer of qPCR instrument | E | SM |
| qPCR validation |  |  |
| Specificity (gel, sequence, melt, or digest) | E | SM |
| For SYBR Green I, Cq of the NTC | E | ∅ |
| Calibration curves with slope and y intercept | E | ∅ |
| PCR efficiency calculated from slope | E | ∅ |
| r^2^ of calibration curve | E | ∅ |
| Linear dynamic range | E | ∅ |
| Cq variation at LOD | E | ∅ |
| Evidence for LOD | E | ∅ |
| If multiplex, efficiency and LOD of each assay | E | ∅ |
| Data analysis |  |  |
| qPCR analysis program (source, version) | E | SM |
| Method of Cq determination | E | SM |
| Outlier identification and disposition | E | SM |
| Results for NTCs | E | SM |
| Justification of number and choice of reference genes | E | SM |
| Description of normalization method | E | SM |
| Number and stage (reverse transcription or qPCR) of technical replicates | E | SM |
| Repeatability (intra-assay variation) | E | SM |
| Statistical methods for results significance | E | SM |
| Software (source, version) | E | SM |
| ^a^ All essential information (E) must be submitted with the manuscript. Desirable information (D) should be submitted if available. If primers are from RTPrimerDB, information on qPCR target, oligonucleotides, protocols, and validation is available from that source.  ^b^ FFPE, formalin-fixed, paraffin-embedded; RIN, RNA integrity number; RQI, RNA quality indicator; GSP, gene-specific priming; dNTP, deoxynucleoside triphosphate.  ∅ Information is not available.  SM Information is provided in Supplementary Materials (* if requested). | | |

**Experimental design**

**Definition of experimental and control groups**

The GV298 lentiviral particles containing the *GBP1P1* shRNA sequence were designed. After transfection of lentivirus into W038 ovarian cancer (OC) cells, cells were screened by purinomycin. Finally, *GBP1P1* knockdown stable W038 OC was obtained as experimental group. The negative control group with disordered shRNA were named sh-CTRL W038.

**Number within each group**

The biological replicates were 3 for each group.

**Sample**

**Description**

W038 OC were inoculated with a density of 2×10^6^/ml and cultured with 6-well culture plate, and RNA was extracted after experimental intervention.

**Microdissection or macrodissection**

Fluorescence microscopy (Lecia, Wetzlar, Germany).

**Processing procedure**

Target cells were cultured with 2 μg/ml of puromycin.

**If frozen - how and how quickly?**

No frozen.

**If fixed - with what, how quickly?**

No fixed.

**Sample storage conditions and duration (especially for FFPE samples)**

Total RNA was extracted by TRIzol method immediately after the intervention.

**Nucleic acid extraction**

**Procedure and/or instrumentation**

​ After the culture solution was sucked out, 1ml of Trizol was added to each hole of the 6-well culture plate, and the solution of Trizol cells was blown up and sucked into a 1.5ml dynamic EP tube.

Absorb Trizol into 1.5ml dynamic EP tube (apply dynamic gun tip)

Let stand at room temperature for 5 minutes;

Add 0.3ml of chloroform, cover tightly and mix manually for 15 seconds;

2 - 3 minutes for the temperature;

Low temperature centrifuge 4℃, 12000g centrifuge for 10 minutes;

Approximately 0.4ml of the supernatant is transferred to a 1.5ml dynamic EP tube;

Add 0.5ml of isopropyl alcohol, mix well and let stand at room temperature for 10 minutes;

Low temperature centrifuges, 10,000 g centrifuges for 10 minutes;

Discard the supernatant, add 1ml 75% ethanol (prepared with DEPC treated water) and mix well;

Low temperature centrifuge 4℃, 7000g centrifuge for 5 minutes;

Discard the supernatant and dry at room temperature for 10 minutes;

DEPC treated water is dissolved in 20ul and stored at -80℃;

The total RNA concentration of the sample was detected using an ultraviolet spectrophotometer.

**Name of kit and details of any modifications**

RNA was obtained from W038 OC samples using TRIzol® reagent.

**Source of additional reagents used**

Sigma (Sigma Aldrich (Shanghai) Trading Co., LTD) and Solarbio (Beijing Solaibao Technology Co., LTD).

**Details of DNase or RNAse treatment**

RNA was obtained from W038 OC samples using TRIzol® reagent and reverse-transcribed with the FastKing gDNA Dispelling RT SuperMix (KR118-02, TIANGEN). All experimental procedures were performed as specified in the kit instructions.

**Contamination assessment (DNA or RNA)**

OD_260/280_ values of RNA samples were detected by ultraviolet spectrophotometer, and OD_260/280_ values of all samples were greater than 1.8 and less than 2.

**Nucleic acid quantification**

Nucleic acid had a high absorption peak in the UV region (260 nm), and the concentration of nucleic acid could be calculated according to the apparent molar absorption coefficient.

**Instrument and method**

The RNA to be measured was dropped into the detection area, and the RNA concentration of the sample was detected by ultraviolet spectrophotometer.

**RNA integrity method/instrument**

RNA integrity was monitored by agarose gel electrophoresis, and the 28S/18S of each sample were between 1.7 and 2.1.

**RIN/RQI or Cq of 3’ and 5’ transcripts**

Information was not available.

**Inhibition testing (Cq dilutions, spike or other)**

The target gene dissolution curves of all samples were unimodal.

**Reverse transcription**

**Complete reaction conditions**

All experimental procedures were conducted as specified in the kit instructions (FastKing gDNA Dispelling RT SuperMix, KR118-02, TIANGEN).

**Amount of RNA and reaction volume**

5×FastKing-RT SuperMix 4.0 μl

Total RNA 2.0 μg

RNase Free dH2O up to 20 μl

**Priming oligonucleotide (if using GSP) and concentration**

RNA was reverse-transcribed with the FastKing gDNA Dispelling RT SuperMix (KR118-02, TIANGEN) in this experiment. The kit includes reagent FastKing-RT SuperMix, which contains dNTP Mixture.

**Reverse transcriptase and concentration**

RNA was reverse-transcribed with the FastKing gDNA Dispelling RT SuperMix (KR118-02, TIANGEN). The kit contains Reverse transcriptase and RNase Inhibitor.

**Temperature and time**

42℃ 15 min

95℃ 3min

**Manufacturer of reagents and catalogue numbers**

FastKing gDNA Dispelling RT SuperMix (KR118-02, TIANGEN).

**qPCR target information**

**Gene symbol**

Official Symbol：GBP1P1

**Sequence accession number**

Gene ID: 400759

**Amplicon length**

88.

**In silico specificity screen (BLAST, and so on)**

Information was not available.

**Location of each primer by exon or intron (if applicable)**

Information was not available.

**What splice variants are targeted?**

Information was not available.

**qPCR oligonucleotides**

**Primer sequences**

| Homo Forward LncRNA-GBP1P1 | CGAGGGTCTGGGAGATGTAGAGAAG |
| --- | --- |
| Homo Reverse LncRNA-GBP1P1 | CATGGAAGTGCTGTTCAGGAGGAC |
| Homo Forward GAPDH | ACGGATTTGGTCGTATTGGG |
| Homo Reverse GAPDH | GGGATCTCGCTCCTGGAAG |

**Location and identity of any modifications**

Information was not available.

**qPCR protocol**

**Complete reaction conditions**

2×SuperReal PreMix Plus 10.0 μl

RT Primer Mix 1.2 μl

cDNA template 1.0 μl

RNase Free dH2O up to 20.0 μl

**Reaction volume and amount of cDNA/DNA**

1.0 μl and not less than 100 ng.

**Primer, (probe), Mg2+, and dNTP concentrations**

| category | concentration |
| --- | --- |
| primer | 10 μM |
| Mg2+ | 2 mM |

SuperReal PreMix Plus (SYBR Green)(FP205, TIANGEN) was selected for this study and the kit included dNTP Mixture.

**Polymerase identity and concentration**

SuperReal PreMix Plus (SYBR Green)(FP205, TIANGEN) was selected for this study and the kit included polymerase.

**Buffer/kit identity and manufacturer**

SuperReal PreMix Plus (SYBR Green)(FP205, TIANGEN).

**Additives (SYBR Green I, DMSO, and so forth)**

The premixed reagent contained SYBR Green I.

**Complete thermocycling parameters**

| phase | cycle | temperature | time | content | Fluorescence signal acquisition |
| --- | --- | --- | --- | --- | --- |
| predegeneration | 1× | 95 ℃ | 15min | predegeneration | No |
| PCR reaction | 40× | 95 ℃ | 10sec | denaturation | No |
|  |  | 60-66℃^△^ | 20-32 sec | Annealing/ extension | Yes |

**Manufacturer of qPCR instrument**

Real-time Fluorescence quantitative PCR Instrument, Hangzhou Bori Technology Co., LTD., FQD-96C.

**qPCR validation**

**Specificity (gel, sequence, melt, or digest)**


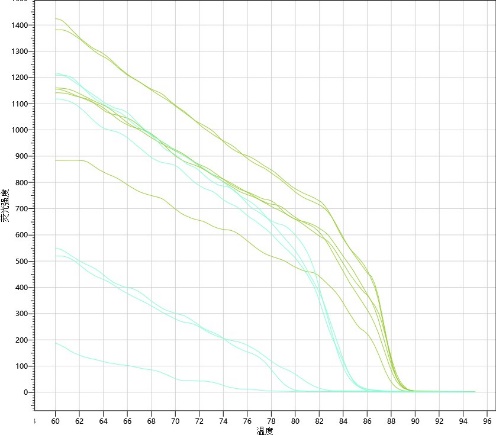

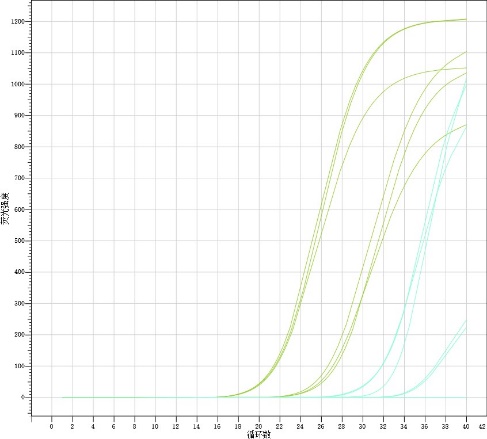


Solution curve Amplification curve

**Data analysis**

**qPCR analysis program (source, version)**

Roche Group LightCycler96.

**Method of Cq determinationCq**

Cq method=15.

**Outlier identification and disposition**

No outliers appear in this experiment.

**Results of NTCs**

No specific peak of NTC appeared.

**Justification of number and choice of reference genes**

The relative expression was calculated using the 2^−△△Ct^ method and estimated relative to GAPDH.

**Description of normalisation method**

All data were normalized by dividing by the mean of the control group before statistical analysis.

**Number and stage (RT or qPCR) of technical replicates**

​During the experimental sample addition process, each sample was repeated with 3 Wells.

**Repeatability (intra-assay variation)**

In this experiment, the difference of CT values between the two holes was less than 0.1.

**Statistical methods for result significance**

For qRT-PCR analysis, the differences among the three groups were evaluated using a one-way analysis of variance (ANOVA) followed by a post hoc test to determine the least significant difference. A significance level of P < 0.05 was considered statistically significant.

**Software (source, version)**

IBM SPSS Statistics 21
