# Supplementary material for: Identification and validation of a prognostic model based on immune-related genes in ovarian carcinoma
Source: PeerJ. 2024 Oct 31;12:e18235. doi: 10.7717/peerj.18235 (PMC11531744; doi:10.7717/peerj.18235)
Supplement: Supplemental Information 2 [file peerj-12-18235-s002.docx]

Supplementary Material

**Supplementary text**

Select immune-related genes (related to Fig. 1C and Table S1)

This gene set contains 458 immune-related genes (IRGs). In the ovaries, these genes are associated with high CD53 / CXCR4 immune cell populations, including separate clusters for adaptive T lymphocytes and Natural Killer (NK) cells, B lymphocytes, and innate immune system, such as monocytes and macrophages. The genes are shown in the table S1.

Select IRGs for clustering (related to Fig. 1 and Table S2)

A total of 243 differentially expressed IRGs (tumor vs normal tissues, FDR<0.05, |Log2(fold-change) | >1) were used for consensus clustering to identify selection of adequate groups. The genes are shown in the table S2.

Untargeted drug sensitivity analysis (related to Table S3, S4 and Fig. S2)

In order to further improve the clinical value of the prognosis models for the drug predictability of OC, we analyzed the commonly used drugs in the clinical treatment of OC. The results showed that oxaliplatin, cisplatin, gemcitabine, ifosfamide, decitabine and irinotecan were more sensitive with higher expression of ACOT13, STAC2, SNRPA1, ZEP1, LEFTY1, SULT1A2 and STX18, and oxaliplatin, gemcitabine, ifosfamide, valrubicin, docelaxel, cyclophospham, epirubicin, 6-mercaptopurine, vincristine and cyclophosphamide was relatively resistant with increasing expression of PYGB, PACSIN3, PI3, FAM120B, GMPR, NSG1 and LYPD6 (p < 0.05).

Table S1. IRGs list

| Gene | Gene | Gene | Gene | Gene | Gene | Gene |
| --- | --- | --- | --- | --- | --- | --- |
| ABI1  ABL2  ABRACL  ACAA1  ACOT9  ACTB  ACTR2  ACTR3  ADA  ADRM1  AHR  AIF1  AKAP13  ALDH2  ALOX5AP  ANXA1  ANXA11  ANXA2  ANXA5  AP1S2  AP2S1  ARF6  ARFGAP3  ARHGDIA  ARHGDIB  ARL4C  ARL6IP5  ARL8B  ARPC1B  ARPC2  ARPC3  ARPC4  ARPC5  ARRB2  ASAH1  ATF5  ATOX1  ATP13A3  ATP1B3  ATP2B1  ATP5F1E  ATP5MD  ATP5MF  ATP5MPL  ATP6AP2  ATP6V0B  ATP6V0D1  ATP6V0E1  ATP6V1B2  ATP6V1F  ATP6V1H  AURKAIP1  B2M  B4GALT1  BASP1  BAX  BAZ1A  BCL2A1  BID  BLOC1S1  BLOC1S2  BLVRB  BNIP3L  BRI3  BTG1  BZW1 | C15orf48  C1orf162  C1QA  CACYBP  CALHM6  CALR  CAP1  CAPG  CAPZA1  CAPZB  CARD16  CASP1  CASP4  CCR7  CD14  CD1C  CD37  CD44  CD48  CD52  CD53  CD58  CD63  CD68  CD74  CD83  CD86  CDC37  CDC42  CDC42EP3  CDC42SE1  CDKN1A  CEBPB  CFD  CFL1  CFLAR  CFP  CHCHD10  CHMP1B  CHMP4B  CIB1  CKLF  CLEC10A  CLEC7A  CLIC1  CLIC2  CMTM6  CNPY3  COPE  CORO1A  CORO1C  COTL1  COX17  COX5A  COX6B1  COX7B  COX8A  CPVL  CREM  CRIP1  CSF2RA  CST3  CSTA  CSTB  CTSB  CTSC | CTSD  CTSH  CTSL  CTSS  CTSZ  CXCL16  CXCL2  CXCL8  CXCR4  CYBA  CYRIA  CYRIB  CYTIP  CYTOR  DAZAP2  DBI  DDX39A  DNTTIP2  DOK2  DPP7  DSE  DUSP1  EFHD2  EHD1  ELF1  ELOF1  EMP3  ENO1  ETS2  EVI2A  EVI2B  EZR  FABP5  FCER1A  FCER1G  FCGR2A  FCGR2B  FCGRT  FCN1  FERMT3  FGL2  FGR  FKBP4  FLNA  FNBP1  FNIP2  FPR3  FTH1  FTL  FXYD5  G0S2  GABARAP  GAPDH  GLA  GLIPR1  GLIPR2  GMFG  GNA15  GNAI2  GNG5  GPAT3  GPR137B  GPR183  GPSM3  GPX1  GPX4 | GRB2  GRINA  GRN  GSTK1  GSTO1  H3-3A  HAVCR2  HCLS1  HCST  HES4  HEXB  HLA-A  HLA-B  HLA-C  HLA-DMA  HLA-DMB  HLA-DPA1  HLA-DPB1  HLA-DQA1  HLA-DQB1  HLA-DRA  HLA-DRB1  HLA-DRB5  HM13  HMGA1  HMOX1  HSBP1  HSP90AA1  HSPA1A  HSPA6  HSPB1  HSPE1  IER3  IFI30  IFNGR1  IFNGR2  IGSF6  IL10RA  IL2RG  IL4I1  INSIG1  IQGAP1  ISG15  ISG20  ITGB2  JAML  JARID2  KYNU  LACTB  LAMTOR2  LAMTOR4  LAP3  LAPTM5  LCP1  LDHA  LGALS1  LGALS2  LGALS3  LGALS9  LGMN  LIMD2  LIMS1  LITAF  LMNA  LPXN  LRRFIP1 | LSP1  LST1  LY86  LY96  LYZ  M6PR  MACROH2A1  MAFB  MAP2K1  MAP2K3  MAP3K8  MARCKS  MBP  MFSD1  MGAT1  MIR22HG  MIR4435-2HG  MMP9  MNDA  MOB1A  MPP1  MRPL18  MS4A6A  MS4A7  MSN  MT-CO1  MT-CO2  MTPN  MYL6  MYO9B  NAA50  NABP1  NAMPT  NAPA  NCOA4  NDUFA13  NDUFB1  NDUFV2  NEAT1  NFKB1  NFKBIA  NINJ1  NOP10  NPC2  NUMB  OAZ1  OLR1  OSBPL8  OSTF1  PABPC1  PCBP1  PDE4DIP  PEA15  PET100  PFN1  PGK1  PGLS  PHACTR1  PIM3  PKIB  PKM  PLAUR  PLEK  PLEKHB2  PLIN2 | POLR2E  POLR2L  POMP  PPIF  PPP1CA  PPP1R18  PPP4C  PPT1  PRDX1  PRR13  PSAP  PSMB3  PSMB9  PSME2  PTP4A2  PTPN1  PTPRC  PTPRE  PYCARD  RAB10  RAB5C  RAB7A  RAB8A  RAB8B  RALA  RAP1A  RAP1B  RBM47  RBX1  REL  RGCC  RGS1  RGS10  RGS2  RHOA  RHOG  RILPL2  RIT1  RNASE6  RNASET2  RNF13  RNF130  RNF145  RNF181  RNH1  S100A10  S100A11  S100A4  S100A6  S100A9  SAMSN1  SAT1  SDCBP  SDS  SEC11A  SEC61B  SERF2  SERPINA1  SERPINB1  SERPINB9  SFT2D1  SH3BGRL3  SLC16A3  SNAP23  SNN  SNX3 | SOD2  SPAG9  SPCS3  SPHK1  SPI1  SPNS1  SQSTM1  SRGN  SSR4  STK4  STMP1  STX11  STX4  SYNGR2  TAGLN2  TALDO1  TANK  TAOK3  TAPBP  TES  THEMIS2  TIMP1  TMEM123  TMEM167A  TMEM273  TMEM9B  TMSB4X  TNFAIP8  TNFRSF1B  TNFSF13B  TNIP1  TPD52L2  TPM3  TPP1  TRAPPC1  TSPO  TUBA1C  TUBB4B  TXN  TXNRD1  TYMP  TYROBP  UBA52  UBE2L3  UBE2R2  UPP1  UQCR11  VAMP8  VASP  VMO1  VPS29  VSIG4  WARS1  WAS  WIPF1  WTAP  YBX1  YWHAH  YWHAZ  ZEB2  ZFAND2A  ZFYVE16  ZNF706 |

Table S2. Differentially expressed IRGs

| \| Gene \|  \| \| --- \| --- \| | logFC | \| Gene \|  \| \| --- \| --- \| | logFC | \| Gene \|  \| \| --- \| --- \| | logFC |
| --- | --- | --- | --- | --- | --- | --- | --- | --- | --- | --- | --- |
| \| ABI1 \| \| --- \| \| ABRACL \| \| ACAA1 \| \| ACOT9 \| \| ACTB \| \| ACTR2 \| \| ADA \| \| ADRM1 \| \| AHR \| \| AIF1 \| \| AKAP13 \| \| ALDH2 \| \| ALOX5AP \| \| ARF6 \| \| ARHGDIB \| \| ARL4C \| \| ARL6IP5 \| \| ARPC1B \| \| ATF5 \| \| ATOX1 \| \| ATP5F1E \| \| ATP6AP2 \| \| ATP6V0B \| \| ATP6V1F \| \| AURKAIP1 \| \| BCL2A1 \| \| BID \| \| BRI3 \| \| C15orf48 \| \| C1QA \| \| CALHM6 \| \| CALR \| \| CAP1 \| \| CAPG \| \| CAPZA1 \| \| CASP4 \| \| CD14 \| \| CD48 \| \| CD52 \| \| CD53 \| \| CD58 \| \| CD74 \| \| CD86 \| \| CDC42 \| \| CDKN1A \| \| CEBPB \| \| CFD \| \| CFLAR \| \| CHCHD10 \| \| CHMP1B \| \| CHMP4B \| \| CIB1 \| \| CLIC1 \| \| CMTM6 \| \| CNPY3 \| \| COPE \| \| CORO1A \| \| COX5A \| \| COX6B1 \| \| COX7B \| \| COX8A \| \| CREM \| \| CRIP1 \| \| CSTA \| \| CSTB \| \| CTSB \| \| CTSD \| \| CTSH \| \| CTSS \| \| CTSZ \| \| CXCL16 \| \| CXCL8 \| \| CXCR4 \| \| CYBA \| \| DSE \| \| EFHD2 \| \| EMP3 \| \| ENO1 \| \| EVI2A \| \| EVI2B \| \| EZR \| | \| 1.086 \| \| --- \| \| 3.242 \| \| -2.070 \| \| -1.035 \| \| 1.853 \| \| 2.127 \| \| 1.011 \| \| 1.677 \| \| 1.856 \| \| 1.640 \| \| -1.954 \| \| -2.679 \| \| 1.455 \| \| 2.751 \| \| 1.670 \| \| 4.387 \| \| 1.485 \| \| 1.767 \| \| 2.127 \| \| -1.253 \| \| 1.409 \| \| 1.137 \| \| 1.001 \| \| 2.159 \| \| 1.276 \| \| 1.399 \| \| 1.526 \| \| -1.530 \| \| 2.448 \| \| 2.593 \| \| 1.210 \| \| 2.073 \| \| 1.160 \| \| 2.713 \| \| 1.574 \| \| -1.060 \| \| 2.324 \| \| 1.320 \| \| 2.635 \| \| 1.769 \| \| 1.088 \| \| 3.348 \| \| 1.418 \| \| 1.060 \| \| -2.171 \| \| 1.698 \| \| -1.080 \| \| -1.670 \| \| 2.991 \| \| 1.665 \| \| 1.484 \| \| 1.804 \| \| 1.444 \| \| 3.257 \| \| 1.786 \| \| 1.008 \| \| 1.021 \| \| 1.739 \| \| 2.464 \| \| 1.540 \| \| 2.108 \| \| -1.974 \| \| -1.341 \| \| -1.238 \| \| 1.834 \| \| 1.146 \| \| 1.893 \| \| -2.052 \| \| 2.227 \| \| 1.305 \| \| 3.428 \| \| 1.776 \| \| 3.701 \| \| 2.312 \| \| -1.627 \| \| 2.099 \| \| -1.657 \| \| 1.564 \| \| 1.293 \| \| 1.908 \| \| 2.249 \| | \| FCER1G \| \| --- \| \| FCGRT \| \| FERMT3 \| \| FGL2 \| \| FKBP4 \| \| FLNA \| \| FPR3 \| \| FTL \| \| FXYD5 \| \| GABARAP \| \| GAPDH \| \| GLIPR1 \| \| GLIPR2 \| \| GNA15 \| \| GNG5 \| \| GPAT3 \| \| GPR137B \| \| GPR183 \| \| GPSM3 \| \| GPX1 \| \| GRB2 \| \| GRINA \| \| GRN \| \| HAVCR2 \| \| HCST \| \| HES4 \| \| HEXB \| \| HLA-B \| \| HLA-C \| \| HLA-DMA \| \| HLA-DMB \| \| HLA-DPA1 \| \| HLA-DPB1 \| \| HLA-QA1 \| \| HLA-QB1 \| \| HLA-DRA \| \| HLA-DRB1 \| \| HLA-DRB5 \| \| HMGA1 \| \| HMOX1 \| \| HSPA1A \| \| HSPB1 \| \| IER3 \| \| IFI30 \| \| IFNGR2 \| \| IGSF6 \| \| IL2RG \| \| IL4I1 \| \| ISG15 \| \| ISG20 \| \| ITGB2 \| \| LAMTOR2 \| \| LAP3 \| \| LAPTM5 \| \| LCP1 \| \| LDHA \| \| LGALS2 \| \| LGALS9 \| \| LSP1 \| \| LST1 \| \| LY86 \| \| LYZ \| \| MAFB \| \| MAP2K3 \| \| MARCKS \| \| MIR22HG \| \| MMP9 \| \| MNDA \| \| MOB1A \| \| MRPL18 \| \| MSN \| \| MT-CO1 \| \| MT-CO2 \| \| MTPN \| \| NABP1 \| \| NAMPT \| \| NAPA \| \| NCOA4 \| \| NDUFA13 \| \| NDUFB1 \| \| NDUFV2 \| | \| 2.972 \| \| --- \| \| -1.627 \| \| 1.134 \| \| 1.008 \| \| 1.451 \| \| -1.419 \| \| 2.227 \| \| 2.479 \| \| 1.306 \| \| -3.502 \| \| 2.371 \| \| -1.003 \| \| 1.193 \| \| 1.132 \| \| 2.061 \| \| -2.251 \| \| 1.152 \| \| 1.214 \| \| 1.997 \| \| 2.849 \| \| 1.009 \| \| 1.327 \| \| 2.028 \| \| 1.518 \| \| 1.783 \| \| 2.053 \| \| -1.105 \| \| 1.383 \| \| 1.424 \| \| 2.506 \| \| 1.726 \| \| 2.286 \| \| 2.489 \| \| 2.307 \| \| 2.547 \| \| 4.845 \| \| 4.437 \| \| 3.623 \| \| 6.627 \| \| -1.215 \| \| -1.113 \| \| 2.073 \| \| 1.936 \| \| -1.969 \| \| 1.085 \| \| 1.000 \| \| 1.932 \| \| 4.391 \| \| 4.519 \| \| -1.359 \| \| 1.520 \| \| 1.318 \| \| 1.511 \| \| 3.220 \| \| 2.551 \| \| 1.238 \| \| 1.129 \| \| 1.172 \| \| 1.382 \| \| 1.111 \| \| 1.989 \| \| 2.998 \| \| 2.444 \| \| 1.314 \| \| 1.741 \| \| -1.073 \| \| 3.186 \| \| 1.358 \| \| 1.834 \| \| 1.143 \| \| 1.003 \| \| 2.131 \| \| 1.539 \| \| 1.639 \| \| -1.389 \| \| -1.002 \| \| -1.532 \| \| 1.691 \| \| -1.881 \| \| -1.868 \| \| -1.724 \| | \| NEAT1 \| \| --- \| \| NOP10 \| \| NUMB \| \| OLR1 \| \| OSTF1 \| \| PABPC1 \| \| PDE4DIP \| \| PEA15 \| \| PFN1 \| \| PKM \| \| PLEK \| \| PLEKHB2 \| \| POLR2L \| \| POMP \| \| PPIF \| \| PPP1CA \| \| PPP4C \| \| PPT1 \| \| PSMB3 \| \| PSMB9 \| \| RAB10 \| \| RAB7A \| \| RALA \| \| RAP1B \| \| RBM47 \| \| RGCC \| \| RGS10 \| \| RGS2 \| \| RHOG \| \| RNASE6 \| \| RNF130 \| \| RNH1 \| \| S100A11 \| \| S100A4 \| \| S100A6 \| \| S100A9 \| \| SDS \| \| SEC11A \| \| SERF2 \| \| SERPINA1 \| \| SERPINB1 \| \| SERPINB9 \| \| SH3BGRL3 \| \| SNAP23 \| \| SOD2 \| \| SPAG9 \| \| SPI1 \| \| SPNS1 \| \| SQSTM1 \| \| SRGN \| \| STMP1 \| \| STX4 \| \| SYNGR2 \| \| TAGLN2 \| \| TAPBP \| \| TES \| \| THEMIS2 \| \| TMEM123 \| \| TMEM167A \| \| TNFSF13B \| \| TPM3 \| \| TRAPPC1 \| \| TSPO \| \| TUBA1C \| \| TUBB4B \| \| TXN \| \| TYMP \| \| TYROBP \| \| UBE2L3 \| \| UBE2R2 \| \| VAMP8 \| \| VASP \| \| VMO1 \| \| VSIG4 \| \| WAS \| \| WTAP \| \| YBX1 \| \| YWHAH \| \| ZEB2 \| \| ZFYVE16 \| \| ZNF706 \| | \| -4.929 \| \| --- \| \| 2.269 \| \| -1.312 \| \| 1.867 \| \| 1.405 \| \| 1.013 \| \| -1.059 \| \| 2.631 \| \| 2.421 \| \| 1.244 \| \| 1.718 \| \| 1.682 \| \| 1.848 \| \| 2.207 \| \| 2.301 \| \| 2.582 \| \| 1.791 \| \| 1.811 \| \| 2.017 \| \| 1.024 \| \| 1.983 \| \| 1.485 \| \| 1.650 \| \| -2.573 \| \| 2.832 \| \| 2.994 \| \| 2.176 \| \| -2.581 \| \| 1.476 \| \| 2.139 \| \| -1.641 \| \| -1.962 \| \| 4.057 \| \| 2.734 \| \| 1.436 \| \| 1.614 \| \| 1.662 \| \| -1.567 \| \| -1.809 \| \| 2.494 \| \| 1.508 \| \| 1.686 \| \| 2.391 \| \| -1.367 \| \| -1.865 \| \| -2.274 \| \| 2.438 \| \| -2.167 \| \| -1.341 \| \| 2.382 \| \| 1.899 \| \| -1.636 \| \| 1.749 \| \| 2.012 \| \| 2.402 \| \| 2.651 \| \| 2.227 \| \| 1.481 \| \| 1.160 \| \| 1.654 \| \| 1.018 \| \| 1.569 \| \| 1.311 \| \| 1.301 \| \| 2.694 \| \| 1.714 \| \| 2.199 \| \| 3.335 \| \| 1.368 \| \| 1.322 \| \| 4.647 \| \| 1.436 \| \| 1.004 \| \| 1.402 \| \| 1.447 \| \| -1.148 \| \| 2.034 \| \| 1.516 \| \| -3.257 \| \| -1.632 \| \| -1.400 \| |

Table S3. Drugs approved by the FDA

| Drug name | Drug name | Drug name |
| --- | --- | --- |
| \| METHOTREXATE \| \| --- \| \| 6-THIOGUANINE \| \| 6-MERCAPTOPURINE \| \| Nitrogen mustard \| \| Allopurinol \| \| Actinomycin D \| \| Chlorambucil \| \| Thiotepa \| \| Melphalan \| \| Triethylenemelamine \| \| Dromostanolone Propionate \| \| Acrichine \| \| Fluorouracil \| \| Nandrolone phenpropionate \| \| TESTOLACTONE \| \| Mithramycin \| \| Pipobroman \| \| Cyclophosphamide \| \| Mitomycin \| \| Floxuridine \| \| Hydroxyurea \| \| Uracil mustard \| \| Dexamethasone Decadron \| \| Mitotane \| \| DACARBAZINE \| \| Vinblastine \| \| Acetalax \| \| Cytarabine \| \| Vincristine \| \| Megestrol acetate \| \| tfdu \| \| Procarbazine \| \| Lomustine \| \| Daunorubicin \| \| Daunorubicin \| \| STREPTOZOCIN \| \| Calusterone \| \| Estramustine \| \| Vinblastine \| \| Fluphenazine \| \| Arsenic trioxide \| \| AZACITIDINE \| \| Cladribine \| \| Mithramycin \| \| Asparaginase \| \| Ifosfamide \| \| Acetalax \| \| Fludarabine \| \| Cisplatin \| \| Isotretinoin \| \| Teniposide \| \| Doxorubicin \| \| Fludarabine \| \| Bleomycin \| \| Paclitaxel \| \| DECITABINE \| \| Mitomycin \| \| Bendamustine \| \| Etoposide \| \| Homoharringtonine \| \| Mithramycin \| \| Tegafur \| \| Parthenolide \| \| Dexrazoxane \| \| Tamoxifen \| \| PENTOSTATIN \| \| RAPAMYCIN \| \| Carboplatin \| \| Valrubicin \| \| Idarubicin \| \| Epirubicin \| \| Oxaliplatin \| \| MITOXANTRONE \| | \| Cytarabine \| \| --- \| \| Mitoxantrone \| \| Fludarabine \| \| Imiquimod \| \| Carmustine \| \| Mithramycin \| \| Rapamycin \| \| Clofarabine \| \| Vinorelbine \| \| Topotecan \| \| Gemcitabine \| \| Bisacodyl \| \| Irinotecan \| \| Docetaxel \| \| Depsipeptide \| \| Simvastatin \| \| Raltitrexed \| \| Midostaurin \| \| 7-Ethyl-10-hydroxycamptothecin \| \| Bortezomib \| \| Irofulven \| \| Temsirolimus \| \| Denileukin Diftitox Ontak \| \| Pemetrexed \| \| Vorinostat \| \| Estramustine \| \| Arsenic trioxide \| \| Eribulin mesilate \| \| Gefitinib \| \| Erlotinib \| \| Fulvestrant \| \| Celecoxib \| \| Zoledronate \| \| Belinostat \| \| Lapatinib \| \| Irinotecan \| \| Dasatinib \| \| Everolimus \| \| Pazopanib \| \| Selumetinib \| \| Imatinib \| \| Lapatinib \| \| Nelfinavir \| \| Nilotinib \| \| Olaparib \| \| Ixabepilone \| \| Raloxifene \| \| Abiraterone \| \| Abiraterone \| \| Sunitinib \| \| Afatinib \| \| Pazopanib \| \| Olaparib \| \| Depsipeptide \| \| pralatrexate \| \| Pemetrexed \| \| Bosutinib \| \| Vismodegib \| \| Actinomycin D \| \| Mitomycin \| \| Lenvatinib \| \| Nelarabine \| \| Crizotinib \| \| DAUNORUBICIN \| \| DIGOXIN \| \| ETHINYL ESTRADIOL \| \| Fluorouracil \| \| Nitrogen mustard \| \| Melphalan \| \| 6-Thioguanine \| \| TYROTHRICIN \| \| Vinblastine \| \| Cabozantinib \| | \| Neratinib \| \| --- \| \| Axitinib \| \| Etoposide \| \| Azacitidine \| \| Floxuridine \| \| tepotinib \| \| Trametinib \| \| Palbociclib \| \| Carfilzomib \| \| Homoharringtonine \| \| Ixazomib citrate \| \| Teniposide \| \| Ponatinib \| \| Bleomycin \| \| Paclitaxel \| \| Rapamycin \| \| Teniposide \| \| Simvastatin \| \| Belinostat \| \| Doxorubicin \| \| Vincristine \| \| Pipamperone \| \| Epirubicin \| \| Idelalisib \| \| Topotecan \| \| ARSENIC TRIOXIDE \| \| 6-Mercaptopurine \| \| Docetaxel \| \| Vorinostat \| \| Gefitinib \| \| Clofarabine \| \| Dasatinib \| \| Irinotecan \| \| VINORELBINE \| \| Copanlisib \| \| Vandetanib \| \| Cabozantinib \| \| Panobinostat \| \| brigatinib \| \| Sonidegib \| \| Sonidegib \| \| Vemurafenib \| \| Ibrutinib \| \| Alectinib \| \| ARRY-162 \| \| Dabrafenib \| \| Alectinib \| \| BMN-673 \| \| Bosutinib \| \| Dacomitinib \| \| ABT-199 \| \| BMN-673 \| \| Cobimetinib (isomer 1) \| \| Cobimetinib (isomer 1) \| \| LY-2835219 \| \| IPI-145 \| \| NMS-E628 \| \| LDK-378 \| \| LDK-378 \| \| Encorafenib \| \| Cobimetinib (isomer 1) \| \| LEE-011 \| \| Osimertinib \| \| PF-06463922 \| \| JNJ-42756493 \| \| LOXO-101 \| \| brigatinib \| \| gilteritinib \| \| Acalabrutinib \| \| Sulfatinib \| \| umbralisib \| \| Copanlisib \| |

Table S4. Untargeted drugs approved by the FDA

| Drug name | Drug name | Drug name |
| --- | --- | --- |
| \| Cyclophosphamide \| \| --- \| \| Vinblastine \| \| Ifosfamide \| \| Cisplatin \| \| Doxorubicin \| \| Paclitaxel \| \| DECITABINE \| \| Carboplatin \| | \| Valrubicin \| \| --- \| \| Epirubicin \| \| Oxaliplatin \| \| Topotecan \| \| Gemcitabine \| \| Irinotecan \| \| Docetaxel \| \| Irinotecan \| | \| Vinblastine \| \| --- \| \| Paclitaxel \| \| Doxorubicin \| \| Vincristine \| \| Epirubicin \| \| 6-Mercaptopurine \| \| Docetaxel \| \| Irinotecan \| |

Suppl. fig. S1


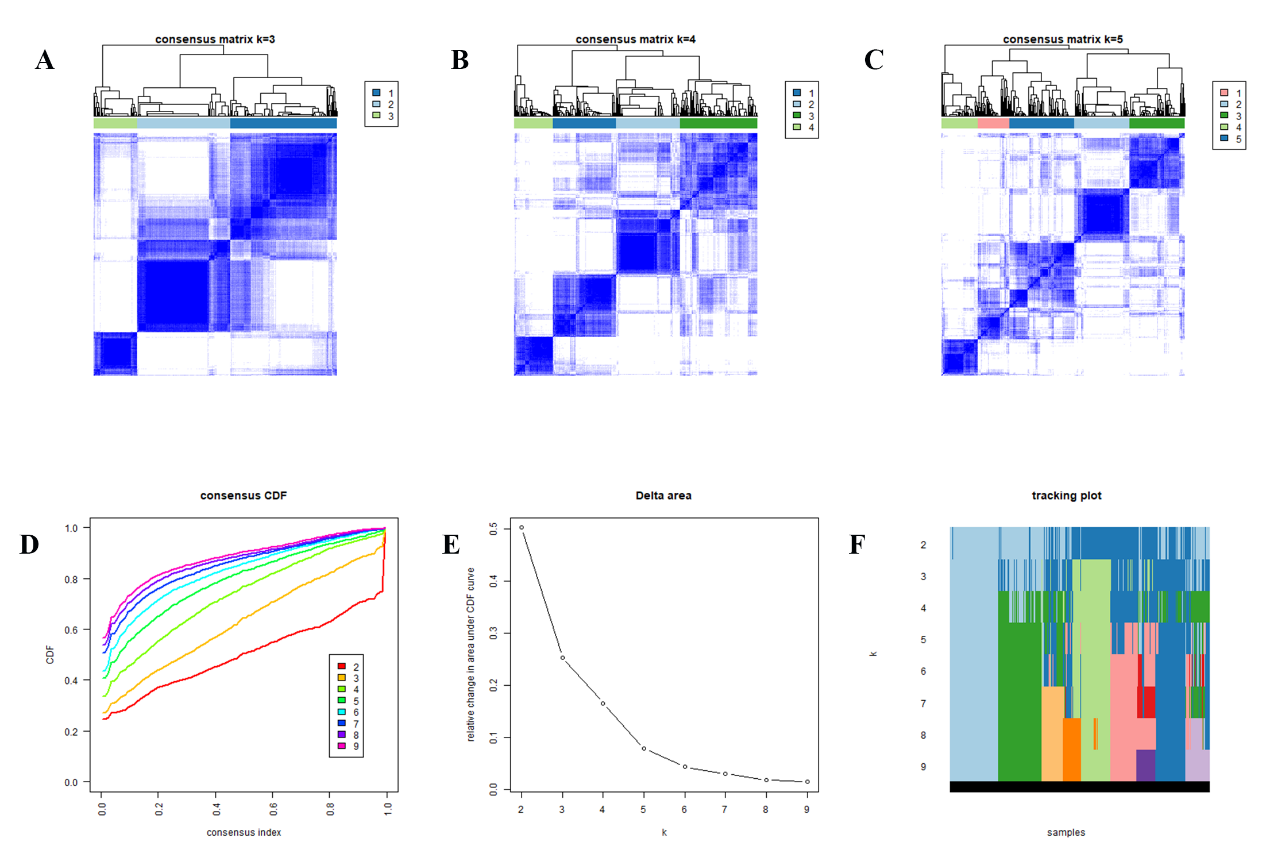


Fig. S1. Consensus clustering for the IRGs in the TCGA dataset. (A) Color-coded heatmap corresponding to the consensus matrix for k = 3 obtained by applying consensus clustering. Color gradients represent consensus values from 0 to 1; white corresponds to 0 and dark blue to 1. (B) Color-coded heatmap corresponding to the consensus matrix for k = 4 obtained by applying consensus clustering. (C) Color-coded heatmap corresponding to the consensus matrix for k = 5 obtained by applying consensus clustering. (D) Consensus clustering cumulative distribution function (CDF) for k = 2 to 9. (E) The Relative change in area under CDF curve for k = 2 to 9. (F) Tracking plot of the consensus clustering analysis.

Suppl. fig. S2


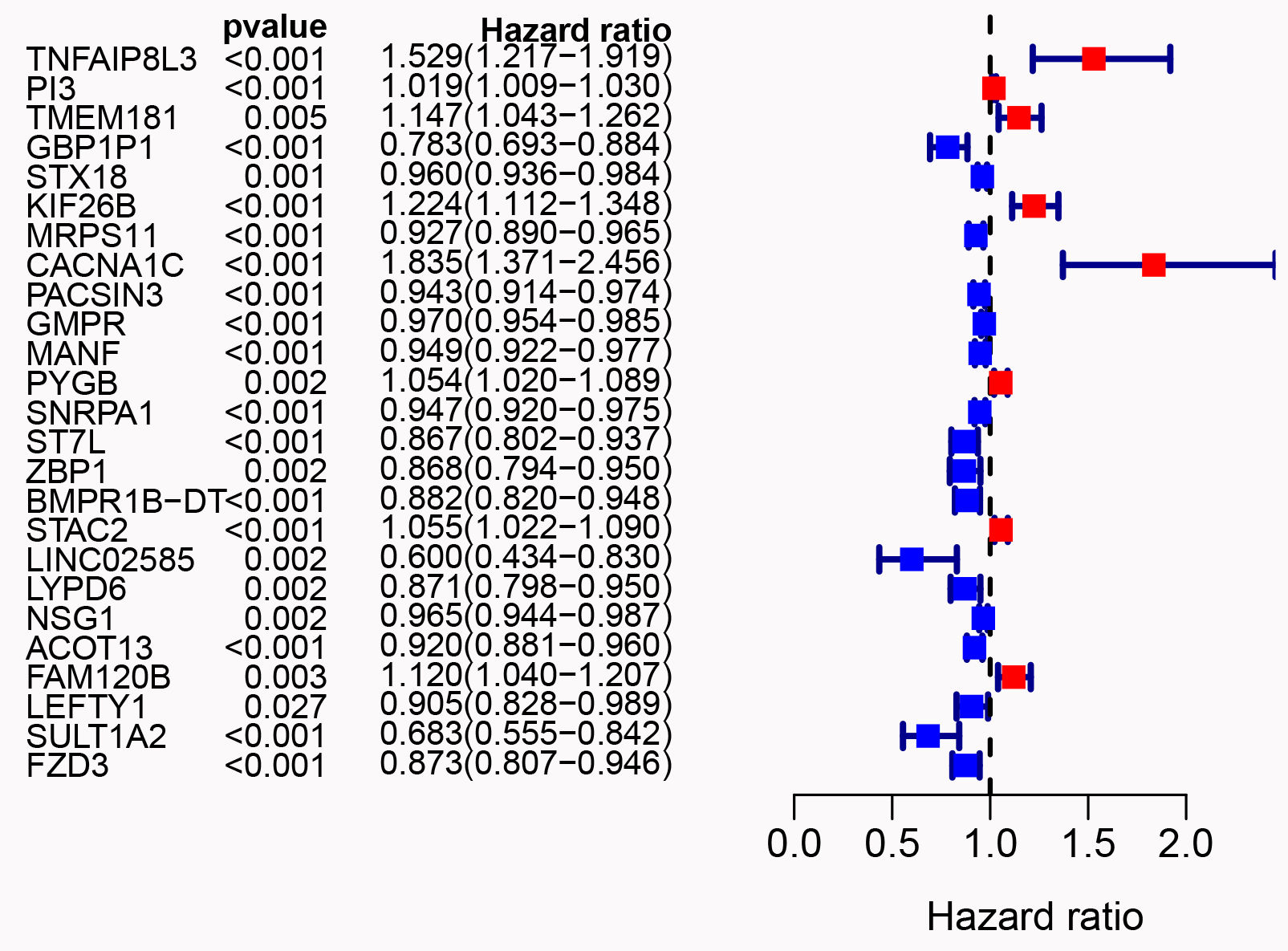


Fig. S2. Univariate Cox regression analysis test of the 25 prognostic IRGs.

Suppl. fig. S3


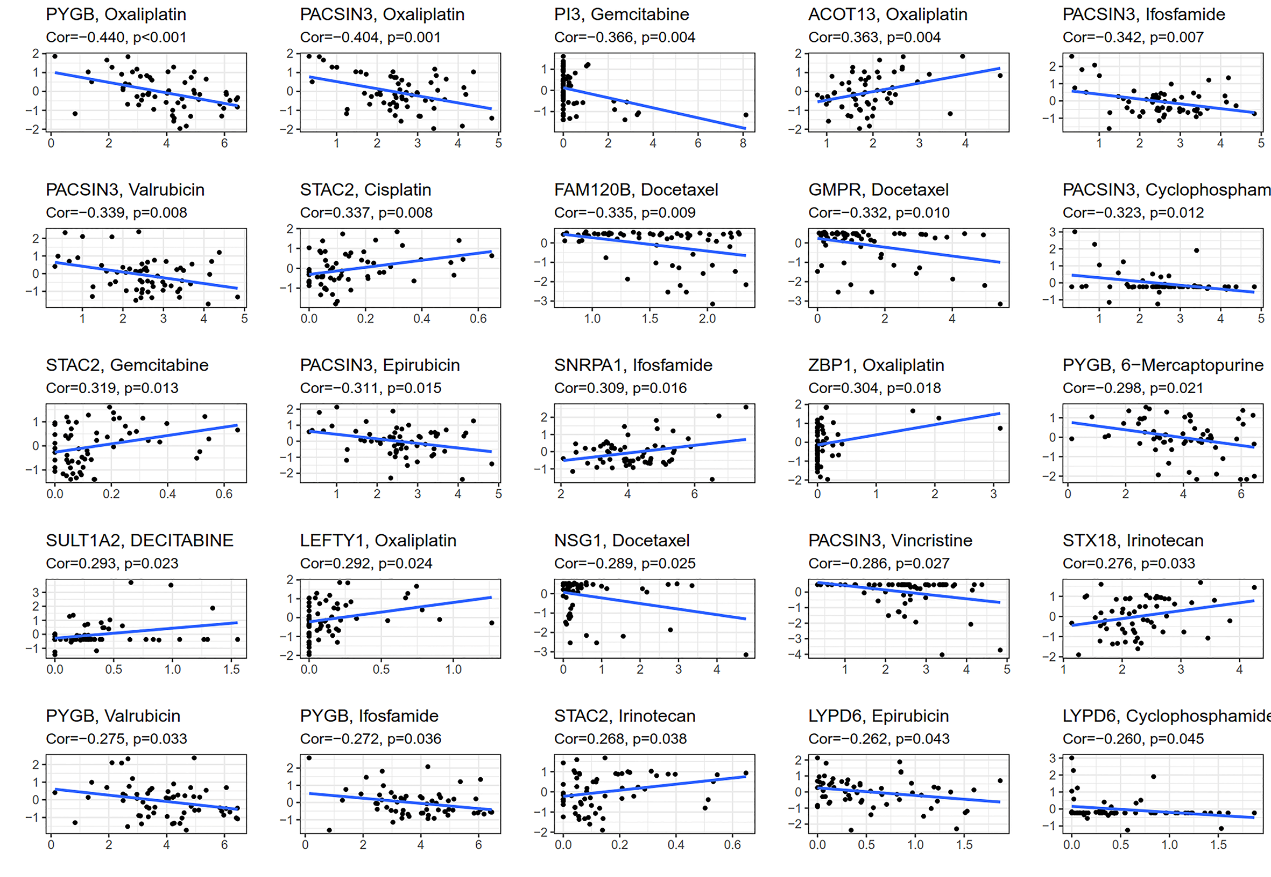


Fig. S3. Gene-untargeted drug sensitivity analysis based on the CellMiner database.

Suppl. fig. S4


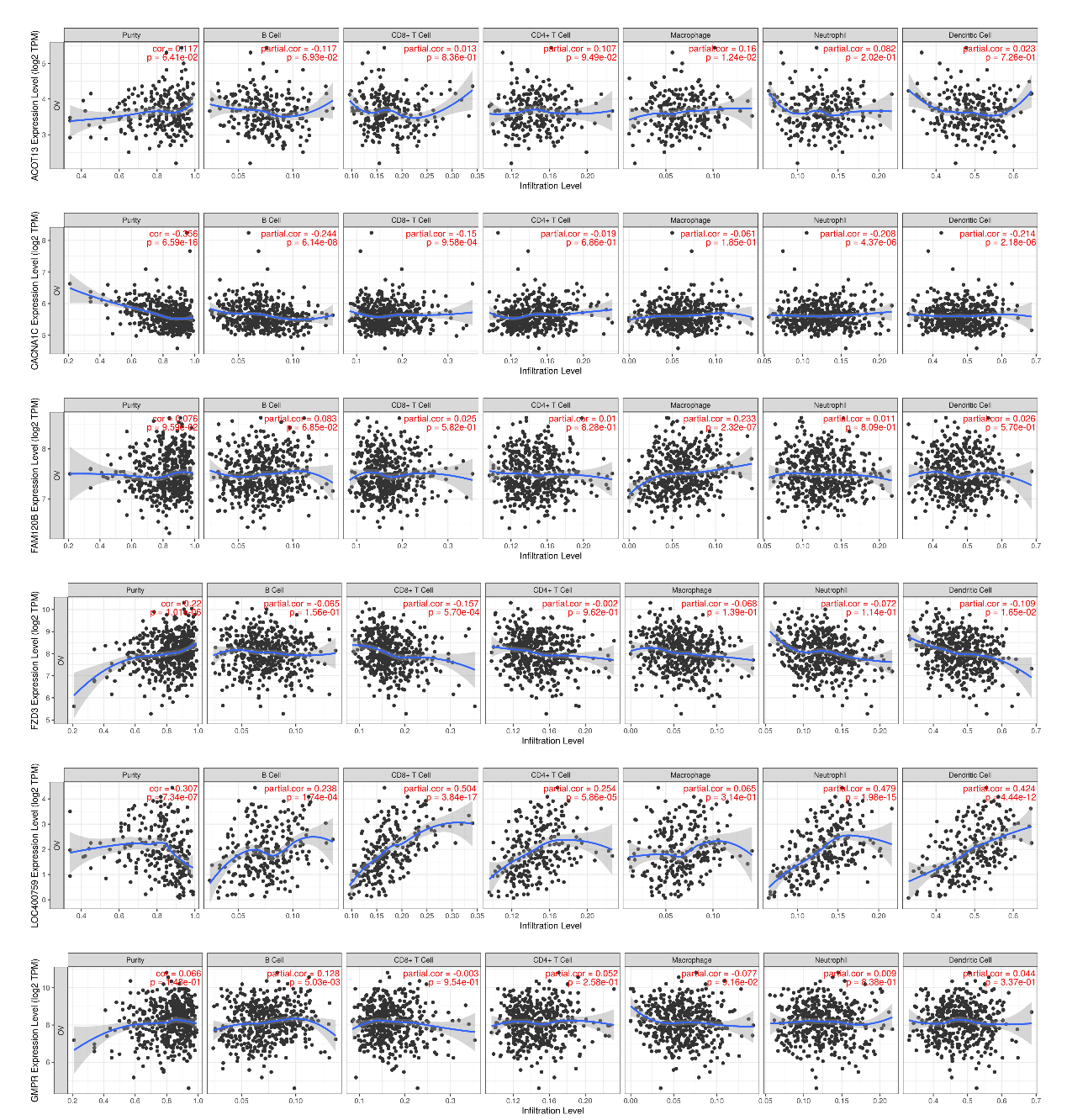


Fig. S4. Correlation analysis of *ACOT13*, *CACNA1C*, *FAM120B*, *FZD3*, *GBP1P1 (LOC400759)*, *GMPR* and immune cell infiltration in OC.

Suppl. fig. S5


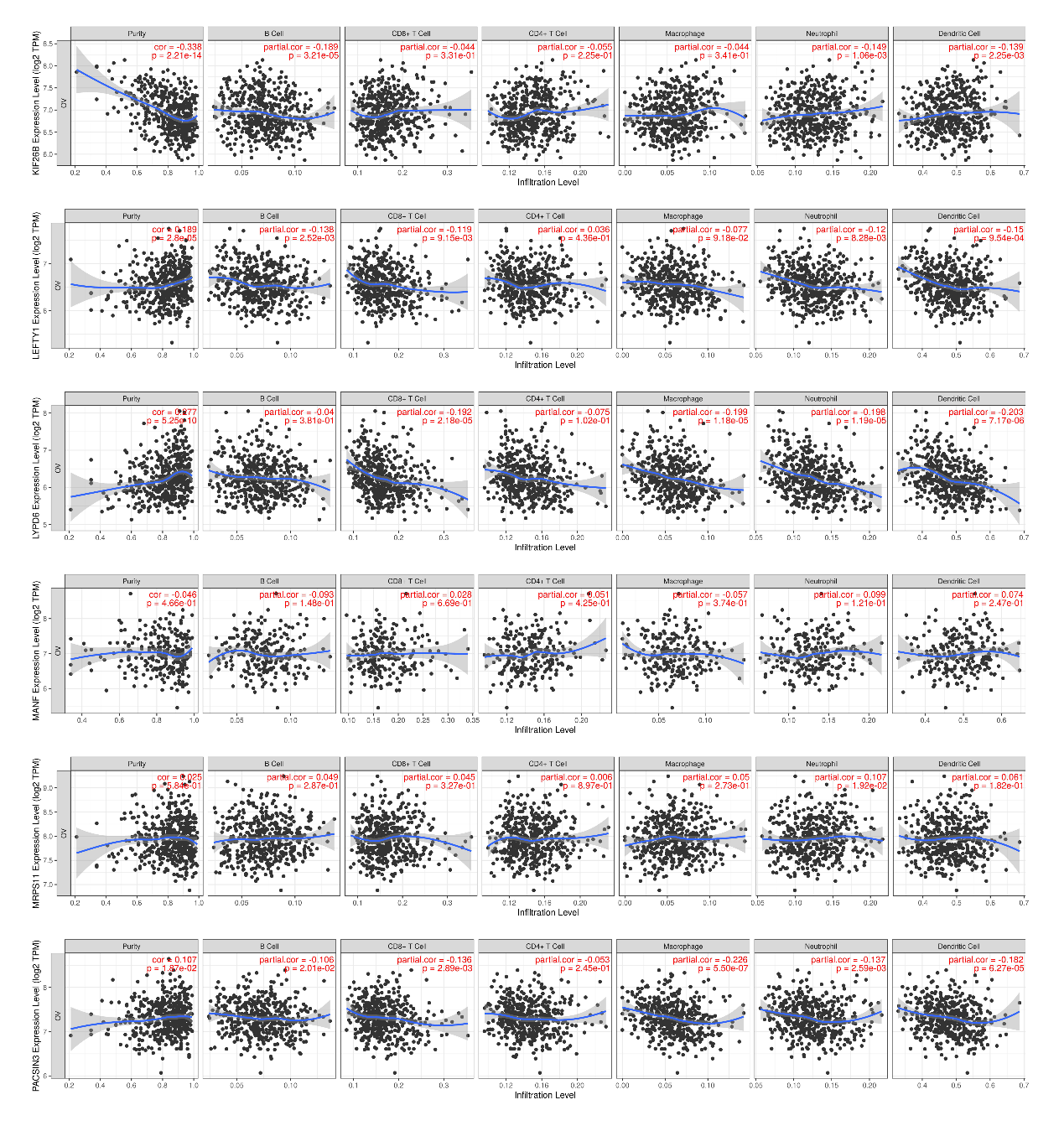


Fig. S5. Correlation analysis of *KIF26B*, *LEFTY1*, *LYPD6*, *MANF*, *MRPS11*, *PACSIN3* and immune cell infiltration in OC.

Suppl. fig. S6


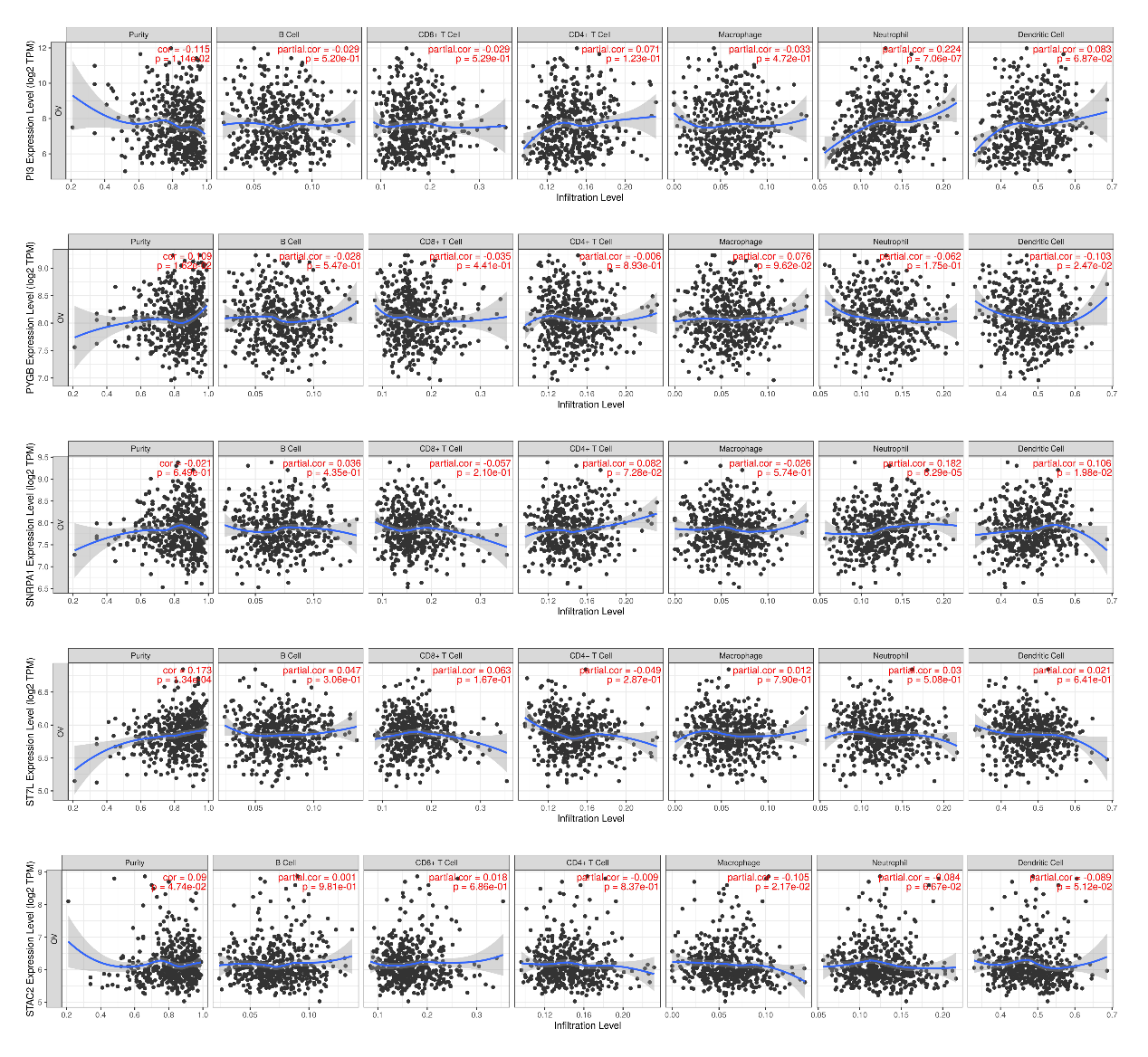


Fig. S6. Correlation analysis of *PI3*, *PYGB*, *SNRPA1*, *ST7L*, *STAC2* and immune cell infiltration in OC.

Suppl. fig. S7


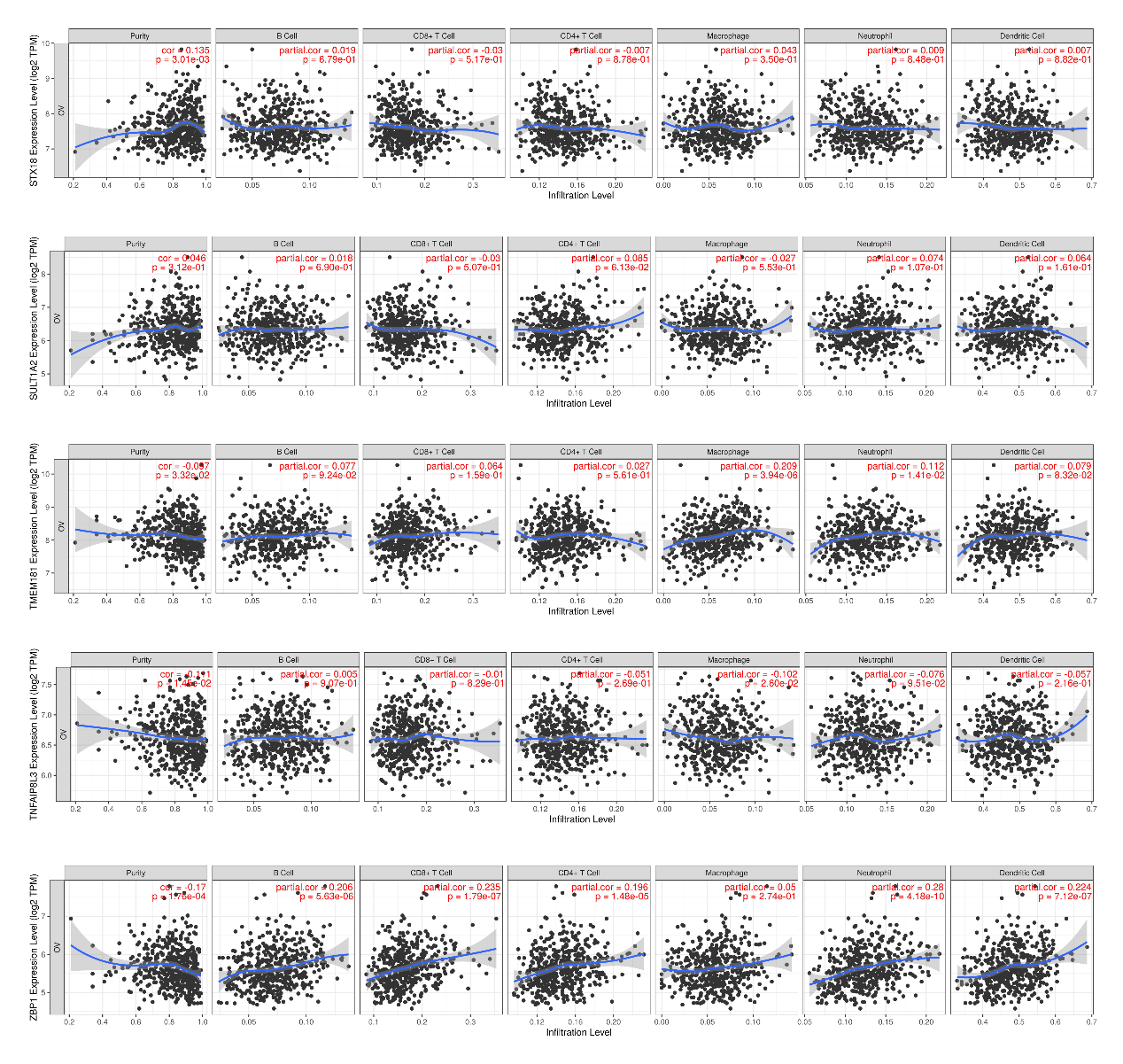


Fig. S7. Correlation analysis of *STX18*, *SULT1A2*, *TMEM181*, *TNFAIP8L3*, *ZBP1* and immune cell infiltration in OC.
